# Senolytic treatment rescues blunted muscle hypertrophy in old mice

**DOI:** 10.1007/s11357-022-00542-2

**Published:** 2022-03-24

**Authors:** Cory M. Dungan, Vandre C. Figueiredo, Yuan Wen, Georgia L. VonLehmden, Christopher J. Zdunek, Nicholas T. Thomas, C. Brooks Mobley, Kevin A. Murach, Camille R. Brightwell, Douglas E. Long, Christopher S. Fry, Philip A. Kern, John J. McCarthy, Charlotte A. Peterson

**Affiliations:** 1 Center for Muscle Biology, University of Kentucky, Lexington, KY USA; 2 Department of Physical Therapy, University of Kentucky, Lexington, KY USA; 3 College of Health Sciences, University of Kentucky, 900 S. Limestone, CTW 445, Lexington, KY 40536 USA; 4 Department of Athletic Training and Clinical Nutrition, University of Kentucky, Lexington, KY USA; 5 Department of Physiology, University of Kentucky, Lexington, KY USA; 6 Present Address: School of Kinesiology, Auburn University, Auburn, AL USA; 7 Present Address: Department of Health, Human Performance, and Recreation, University of Arkansas, Fayetteville, AR USA; 8 Department of Internal Medicine, Division of Endocrinology, University of Kentucky, Lexington, KY USA

**Keywords:** Senescence, Senolytics, Skeletal muscle, Hypertrophy, Anabolic resistance

## Abstract

With aging, skeletal muscle plasticity is attenuated in response to exercise. Here, we report that senescent cells, identified using senescence-associated β-galactosidase (SA β-Gal) activity and p21 immunohistochemistry, are very infrequent in resting muscle, but emerge approximately 2 weeks after a bout of resistance exercise in humans. We hypothesized that these cells contribute to blunted hypertrophic potential in old age. Using synergist ablation-induced mechanical overload (MOV) of the plantaris muscle to model resistance training in adult (5–6-month) and old (23–24-month) male C57BL/6 J mice, we found increased senescent cells in both age groups during hypertrophy. Consistent with the human data, there were negligible senescent cells in plantaris muscle from adult and old sham controls, but old mice had significantly more senescent cells 7 and 14 days following MOV relative to young. Old mice had blunted whole-muscle hypertrophy when compared to adult mice, along with smaller muscle fibers, specifically glycolytic type 2x + 2b fibers. To ablate senescent cells using a hit-and-run approach, old mice were treated with vehicle or a senolytic cocktail consisting of 5 mg/kg dasatinib and 50 mg/kg quercetin (D + Q) on days 7 and 10 during 14 days of MOV; control mice underwent sham surgery with or without senolytic treatment. Old mice given D + Q had larger muscles and muscle fibers after 14 days of MOV, fewer senescent cells when compared to vehicle-treated old mice, and changes in the expression of genes (i.e., *Igf1*, *Ddit4*, *Mmp14*) that are associated with hypertrophic growth. Our data collectively show that senescent cells emerge in human and mouse skeletal muscle following a hypertrophic stimulus and that D + Q improves muscle growth in old mice.

**Supplementary Information:**

The online version contains supplementary material available at 10.1007/s11357-022-00542-2.

## Introduction

Aging is accompanied by a host of physical and metabolic decrements that includes sarcopenia, which is the gradual loss of muscle mass and strength [1, 2]. To slow muscle mass loss, resistance exercise is often employed; however, gains in muscle mass are blunted following resistance exercise of aged individuals when compared to younger adults, a phenomenon known as anabolic resistance [3]. Various mechanisms, such as dysregulated growth signaling [4, 5], altered protein anabolism and catabolism [6], defective autophagy [7], and reduced mitochondrial function [8], have been shown to contribute to anabolic resistance; however, our recent work suggests that an accumulation of senescent cells during muscle remodeling could be a contributing factor [9]. Senescent cells secrete a host of biomolecules including pro-inflammatory cytokines, growth factors, and proteases known as the senescent-associated secretory phenotype (SASP) [10, 11]. The SASP can blunt mTORC1-mediated growth signaling [12], elevate protein catabolism [13], and dysregulate cytoskeletal remodeling [14], which may result in blunted muscle growth and contribute to anabolic resistance in aged muscle.

Various ergogenic aids, such as metformin [15] and testosterone [16], have been utilized to help facilitate exercise adaptation in aged individuals. Unfortunately, these interventions either do not enhance muscle growth [15] or have health risks that outweigh any benefits to gains in muscle mass [17]. Senolytics are an emerging class of pharmaceuticals that preferentially kill senescent cells by inducing apoptosis through the inhibition of anti-apoptotic machinery [18–20]. Specifically, the senolytic cocktail of dasatinib and quercetin (D + Q) has been used to extend lifespan [21], improve physical function and healthspan [9, 21], and augment skeletal muscle repair [22]. Recently, we showed that D + Q was sufficient to reduce senescent cell burden and enhance muscle regeneration following injury in old mice [9]. Furthermore, D + Q treatment led to reduced inflammatory and SASP gene expression and upregulation of genes required for glycolysis and muscle contraction 7 days following muscle injury [9]. Our data, combined with others showing beneficial effects of senolytics on skeletal muscle repair [22], strongly suggest that senolytics can improve the hypertrophic response to resistance exercise with aging.

Our laboratory [9, 23] and others [24, 25] report little-to-no senescent cells in resting muscle from old mice, humans, and non-human primates, along with no difference in senescence marker gene expression [26] or effect of senolytics on resting muscle mass and size [9, 27]; however, senescent cells do accumulate in muscle following injury [28–31] and there appear to be sex-specific effects of systemic senescent cell ablation on sarcopenia [32]. The goals of this study were therefore to (1) quantify the senescent cell burden in skeletal muscle from young and old mice in response to a hypertrophic stimulus and (2) determine if treating old mice with D + Q would augment gains in muscle mass following mechanical overload (MOV) associated with reduced senescent cell burden. We hypothesized that senescent cells would appear in muscle in response to exercise, and that D + Q treatment would have a beneficial effect on hypertrophy in aged muscle.

## Methods

### Human subjects

Muscle biopsies and associated phenotyping data from a total of 18 de-identified men (*n* = 10) and women (*n* = 8) from the University of Kentucky are presented and summarized in Supplemental Table 1. The subjects were generally healthy, non-diabetic, and both lean and obese, and had no chronic medical conditions and were not taking any medications that would interfere with muscle function. Subjects had a pre-exercise biopsy taken, performed a session of whole-body resistance exercise to exhaustion, and then had muscle biopsies taken approximately 14 days later. The exercise bout consisted of three lower body (squat, leg press, and leg extension) movements and one upper body (lat pulldown) movement at approximately 70–80% of one repetition max (1RM). Three sets of eight repetitions were completed as well as a fourth set to failure with 90–120 s of rest given between each set. Vastus lateralis muscle biopsies were obtained as described previously [15, 33] and samples for immunohistochemical analysis were mounted in tragacanth gum mixed with OCT and quickly frozen in liquid nitrogen–cooled isopentane and stored at − 80 °C until sectioning. All protocols were approved by the Institutional Review Board of the University of Kentucky, Lexington, KY, USA, and performed in accordance with the standards set forth by the Declaration of Helsinki.

### Animals

Adult (5–6-month) and old (23–24-month) male C57BL/6 J mice were purchased from the Jackson Laboratory (Bar Harbor, ME) and the NIA (National Institute on Aging, Bethesda, MD), respectively. All animals were housed in Division of Laboratory Animal Resources at University of Kentucky until they reached the expected age before experiments started. They were housed in a temperature- and humidity-controlled room and maintained on a 14:10-h light:dark cycle with food and water ad libitum. Mice were euthanized by IP injection of pentobarbital sodium followed by cervical dislocation. All experimental procedures performed in this study were approved by the University of Kentucky Institutional Animal Care and Use Committee.

### Synergist ablation surgery (mechanical overload)

Animals were subjected to bilateral synergist ablation surgery to induce hypertrophy of the plantaris muscle as previously described [34, 35]. Briefly, following anesthetization with 5% isoflurane, 1/4–1/3 of the soleus and the gastrocnemius muscles were surgically excised via an incision on the dorsal aspect of the hind limb. Particular attention was made to ensure neural and vascular supply to the plantaris muscle remained intact and undamaged. Sham surgeries were performed as controls, in which the skin was opened without removal of the gastrocnemius and soleus muscles. Plantaris muscle was collected at 7 and 14 days after the surgery. Control plantaris muscles (sham operated) were collected from mice on every collection day.

### Senolytic administration

Mice were administered a senolytic cocktail containing 5 mg/kg dasatinib (D-3307, LC Labs, Woburn, MA) and 50 mg/kg quercetin (Q4951, Sigma-Aldrich, St. Louis, MO) in a similar manner as described by Xu et al. [21] and consistent with a previous study by our laboratory [9]. Briefly, 7.5 mg of dasatinib and 75 mg of quercetin were dissolved in 5 mL of 10% polyethylene glycol 400 (PEG 400; 202398, Sigma-Aldrich). We chose this volume of PEG 400 because it allowed us to gavage a 30–45 g mouse with 100–150 μL of senolytic, which is much less than the approximate stomach volume of 400 μL in adult mice [36]. Senolytics or vehicle (10% PEG) was administered on day 7 and day 10 of the 14-day mechanical overload protocol (Fig. 5a) using 20-gauge disposable polypropylene feeding tubes (FTP-20–30, Instech, Plymouth Meeting, PA). We chose a hit-and-run approach based on the work from the Kirkland laboratory and these time points because we previously showed that SA β-Gal + cells appear as early as 7 days in a muscle injury model [9]. Furthermore, MOV places a constant stress on the muscle such that senescent cells would continue to appear after the initial D + Q gavage and it is unlikely that D + Q kills all of the senescent cells after a single treatment.

### Senescence-associated β-galactosidase (SA β-Gal) staining

The SA β-Gal staining protocol used was adapted from a previously published protocol by our laboratory [23]. Briefly, freshly cut 8 μm muscle sections were fixed in 0.5% glutaraldehyde for 5 min at room temperature and washed in PBS. After washing, sections were incubated in freshly made staining solution that contained the following: 1 mg/mL X-gal in DMF, 5 mM potassium ferrocyanide, 5 mM potassium ferricyanide, 5 M sodium chloride, 1 M magnesium chloride, and 0.2 M citric acid/Na phosphate buffer pH 6.0 ± 0.05. Muscle sections were incubated in staining solution for 72 h at 37 °C in a dark hybridization oven, with fresh solution added every 24 h. Afterwards, sections were washed in PBS for up to 24 h to remove salt crystals (this does not affect staining) and then cover slipped using a 1:1 ratio of PBS and glycerol.

### Immunohistochemistry (IHC)

IHC analyses to measure fiber type distribution, mean muscle fiber cross-sectional area (CSA), and fiber type-specific CSA were performed as previously described by our laboratory [37, 38]. To summarize, 8 μm muscle sections were incubated overnight in a cocktail of concentrated myosin heavy chain (MyHC) primary antibodies from the University of Iowa Developmental Studies Hybridoma Bank for MyHC 1 (1:200; BA.D5) and MyHC 2a (1:200; SC.71), and dystrophin (1:200; ab15277, Abcam, Cambridge, UK) to label the fiber border. The following day, sections were washed in PBS and incubated in appropriate fluorescent secondary antibodies for 90 min. Type 2x + 2b fibers are unstained (black). Finally, sections were washed in PBS and mounted in PBS:glycerol.

To quantify p21 + cells, 8 μm muscle sections were fixed in 4% paraformaldehyde and washed in PBS, and then endogenous peroxidases were quenched with 3% H_2_O_2_, each for 10 min. Sections were blocked in 2% bovine serum albumin (BSA) that contained 0.1% Triton X-100 (blocking buffer) for 1 h. Sections were then incubated in primary antibodies against p21 (1:200; ab109199, Abcam) and laminin (1:100; MA5-24,656, Invitrogen) overnight in blocking buffer. The following day, sections were washed in PBS and incubated in fluorescent secondary antibodies, each for 90 min. Specifically, p21 required incubation with anti-Rb biotin (1:1000; 111–064-003, Jackson ImmunoResearch, West Grove, PA) diluted in blocking buffer for 75 min, followed by streptavidin-HRP (1:500; SA10001, Invitrogen) diluted in PBS for 75 min and then TSA AF488 (1:500; B40953, Invitrogen) diluted in DAPI staining solution (1:10,000 in PBS; D3571, Invitrogen) for 15 min. During the streptavidin-HRP step, anti-Ms IgG1 AF594 (1:100; A-21125, Invitrogen) was added to label laminin. Sections were washed in PBS between each secondary step.

### Image capture and analysis

An Axio Imager M1 upright microscope equipped with ZEN software (Zeiss, Oberkochen, Germany) was used to capture stitched images of the entire muscle cross-sections for cell counts. Images were minimally post-processed for color balance, contrast, and brightness. Mean myofiber CSA and fiber type-specific CSA were quantified using MyoVision automated analysis software [39]. The dystrophin labeled image was used as the reference and myofiber CSA was automatically determined by MyoVision’s region of interest algorithm. Myofibers with CSAs below 300 µm^2^ and above 6,000 µm^2^ were excluded from the analysis. SA β-Gal + and p21 + cells were manually counted by a blinded, trained technician and expressed relative to the total area of the muscle section. Regions of the cross-section that appeared to be folded on top of another region or appeared to be damaged during cryosectioning were manually excluded from the analysis.

### RNA isolation and RNA sequencing

RNA was isolated from plantaris muscles from vehicle- and D + Q-treated mice (*n* = 5/group) 14 days following MOV using the Qiagen miRNeasy® Mini Kit (Hilden, Germany). RNA concentration was assessed using a Nanodrop 2000 (Thermo Fisher) and RNA integrity was quantified using an Agilent Bioanalyzer 2100 (Agilent Technologies, Santa Clara, CA). Five hundred nanograms of high-quality RNA (RIN > 8.0) was then sent to Novogene (Beijing, China) for sequencing. RNA-seq analyses were performed using *Partek® Flow®* software, v10.0 (St. Louis, MO). Pre-alignment quality control was completed using the default QA/QC tool. Alignment of sequencing reads to the mouse genome (GRCm39) using the splice-aware program STAR (v2.7.8a). Gene counts were quantified using Partek E/M against transcriptome release 103 and a minimum expression cutoff of 10 counts was used to filter out low expression genes. Differential gene expression was analyzed using DESeq2 (FDR<0.05) (v3.5). Gene set over-representation analysis was performed using Consensus Path DB software using default settings and FDR<0.05 genes [40, 41].

### Statistical analysis

All human data were analyzed using a paired *t*-test. All mouse data were analyzed by two-way ANOVA, in which animal age and time point were main effects. Post hoc analysis was performed with the Holm-Šídák method because the Šídák modification provides more power than the Holm step-down method alone, which is more powerful than other methods such as Tukey and Bonferroni. Significance was set as *p* < 0.05. Data points that were > 2 standard deviations away from the mean were considered outliers. Statistics were performed using Prism 9 for Mac (GraphPad Software, San Diego, CA).

## Results

### Senescent cells manifested in human muscle following a bout of resistance exercise

**Fig. 1:**
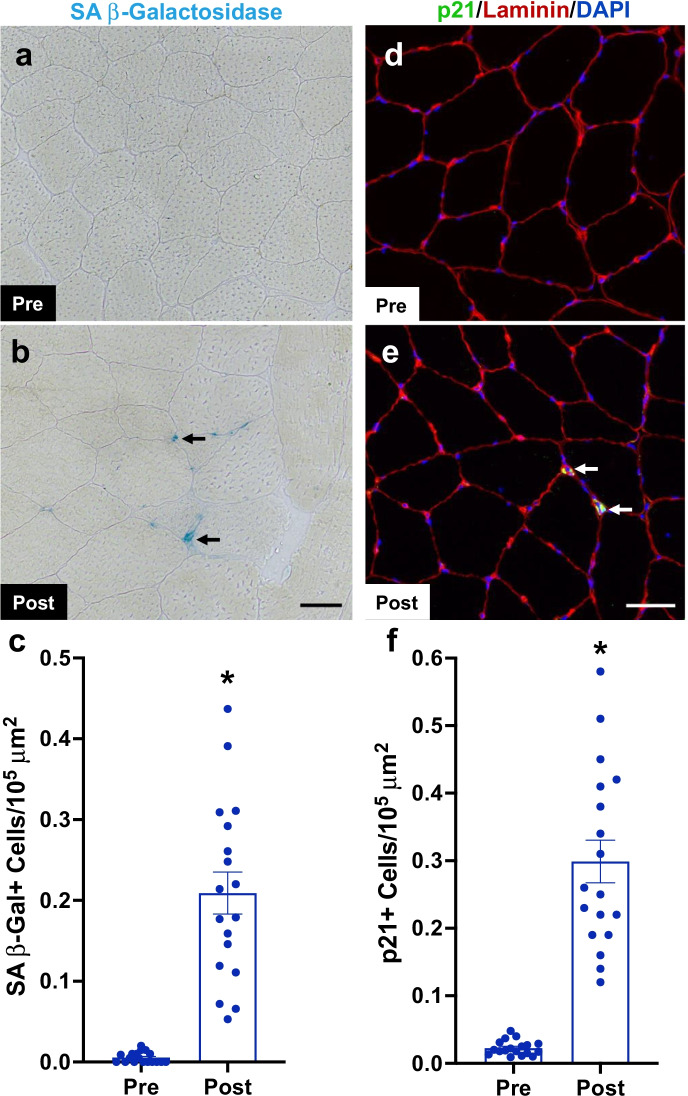
Senescent cell accumulation in human skeletal muscle following a bout of resistance exercise. (**a**), (**b**) Representative images for senescence-associated beta-galactosidase (SA β-Gal) staining of the vastus lateralis sections prior to and 14 days after a bout of whole-body resistance exercise. (**c**) Quantification of SA β-Gal + cells per 10^5^ μm^2^ pre- and post-exercise. (**d**), (**e**) Representative images of p21 immunohistochemistry in vastus lateralis sections prior to and 14 days after a bout of whole-body resistance exercise. (**f**) Quantification of p21 + cells per 10^5^ μm^2^ pre- and post-exercise. *N* = 18. *Significance versus pre-exercise

To see if the appearance of senescent cells following resistance exercise is a feature of human muscle adaptation, a cohort of volunteers (20–39 years old; Supplemental Table 1) had a resting vastus lateralis muscle biopsy prior to exercise, then underwent an exhaustive session of resistance exercise with a second muscle biopsy taken approximately 14 days later. Consistent with our previous report [23], there were essentially no SA β-Gal + (Fig. 1a–c) or p21 + (Fig. 1d–f) cells detectable in resting muscle biopsies; however, senescent cells were increased 14 days after the exercise bout (Fig. 1c, f). Of note, in cells located outside of the muscle fiber, p21 was almost exclusively located in the cytoplasm. Cytoplasmic p21 exerts anti-apoptotic functions [42, 43], which could contribute to an elevation of senescent cell anti-apoptotic pathways (SCAPs) activated in senescent cells [44]. Although rare, we observed nuclearly localized p21 almost exclusively within the muscle fiber (laminin) border in response to a hypertrophic stimulus. This distinction is important given that p21 is indispensable for muscle stem cell differentiation and fusion [45, 46] by interacting with nuclear transcription factors [47]. Representative images are shown in Supplemental Fig. 1.

### Muscle hypertrophy was blunted in old mice

**Fig. 2:**
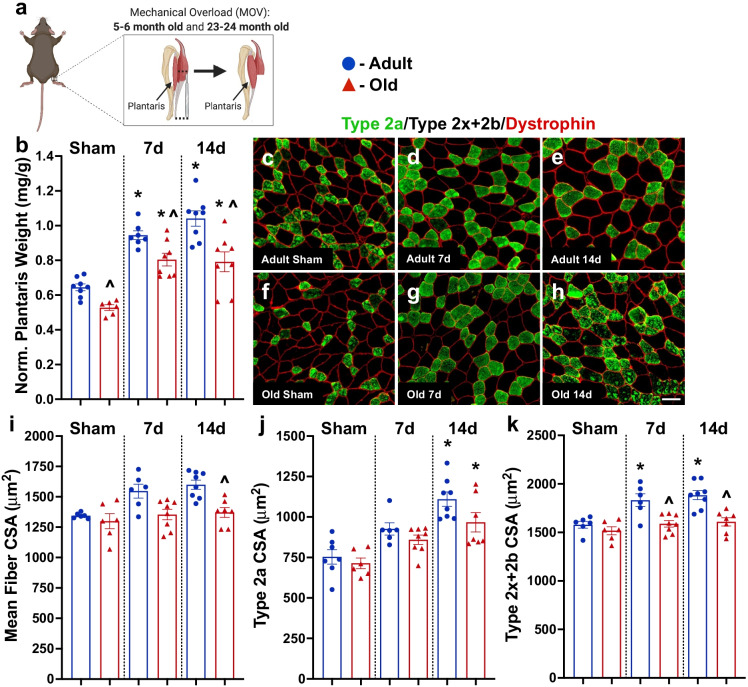
Blunted hypertrophy in old mice in response to mechanical overload (MOV). (**a**) Study design schematic for the synergist ablation-induced MOV time course of the plantaris muscle in adult and old mice. (**b**) Normalized muscle weight in adult (blue circles) and old (red triangles) mice following sham surgery (controls) and 7 and 14 days of MOV induced by synergist ablation surgery. Representative images of fiber-type specific CSA in adult (**c–e**) and old (**f–h**) mice. (**i**) Mean, (**j**) type 2a, and (**k**) type 2x + 2b muscle fiber CSA. *N* = 6–8/group. *Significance versus sham controls for a given age group. ^Significance versus adult mice for a given time point

Both adult and old mice showed an increase in senescent cells following injury; however, in muscle, these cells preferentially persist in the old animals, contributing to a lower muscle regenerative response [9]. These findings, together with the results described above in humans, led us to hypothesize that persistent senescent cell accumulation may contribute to anabolic resistance observed with old age. To test this hypothesis, mice were subjected to synergist ablation surgery where removal of a portion of the gastrocnemius and soleus muscles in the hindlimb results in mechanical overload (MOV) of the plantaris. We examined muscle mass and fiber cross-sectional area (CSA) after 7 and 14 days of MOV in adult (5–6-month-old) and old (23–24-month-old) mice (experimental design, Fig. 2a). Old mice were significantly heavier than adult mice (*p* < 0.05; 33.0 ± 2.6 g vs. 29.8 ± 1.8 g, respectively), and both adult and old mice had heavier muscles following MOV when compared to age-matched sham controls, but plantaris muscle hypertrophy was blunted in old mice when compared to young (Fig. 2b; Supplemental Fig. 2a). Mean muscle fiber CSA was significantly larger following 7 and 14 days of MOV in adult mice when compared to age-matched sham controls (Fig. 2c–e, i), whereas old mice did not display larger muscle fibers when compared to controls at these same time points (Fig. 2f–h, i). We then examined fiber-type specific CSA and observed that both adult and old mice had significantly larger type 2a muscle fibers at 7 and 14 days of MOV compared to sham (Fig. 2j), whereas only adult mice had larger type 2x + 2b fibers at 7 and 14 days (Fig. 2k). There was no difference in fiber-type distribution between groups (Supplemental Fig. 2b-c).

### Senescent cells accumulated to higher levels in old compared to adult mice following MOV

**Fig. 3:**
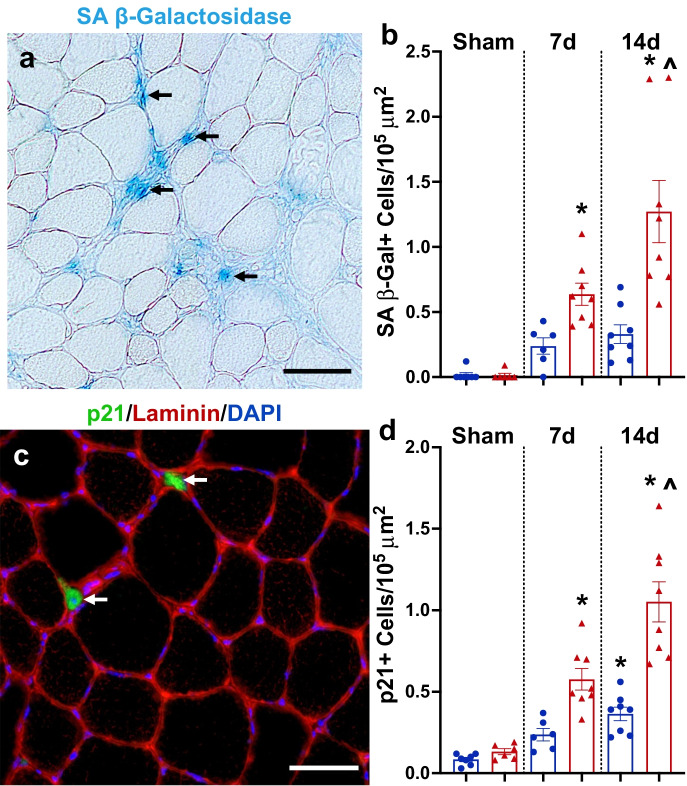
Senescent cells accumulated in skeletal muscle following mechanical overload (MOV). (**a**) Representative image for senescence-associated beta-galactosidase (SA β-Gal) staining 14 days following MOV in an old mouse. (**b**) Quantification of SA β-Gal + cells per 10^5^ μm^2^ in adult (blue circles) and old (red triangles) mice following sham surgery (controls) and 7 and 14 days of MOV induced by synergist ablation surgery. (**c**) Representative image of p21 immunohistochemistry in the plantaris 14 days following MOV in an old mouse. (**d**) Quantification of p21 + cells per 10^5^ μm^2^ in adult (blue circles) and old (red triangles) mice following sham surgery (controls) and 7 and 14 days of MOV induced by synergist ablation surgery. *N* = 6–8/group. *Significance versus sham controls for a given age group. ^Significance versus adult mice for a given time point

In sham mice from adult and old mice, there were very few senescent cells, with no difference in SA β-Gal + (Fig. 3a, b) or p21 + (Fig. 3c, d) senescent cell burden. Following 7 and 14 days of MOV, the senescent cell burden was significantly higher in old mice versus sham (Fig. 3b, d). At 14 days, senescent cell abundance was significantly higher in old compared to adult mice (Fig. 3b, d). These data show that senescent cells are elevated in response to MOV-induced muscle hypertrophy in adult and old mice, but to a higher level in muscle from old mice.

### D + Q improved gains in muscle mass following 14 days of MOV in old mice

**Fig. 4:**
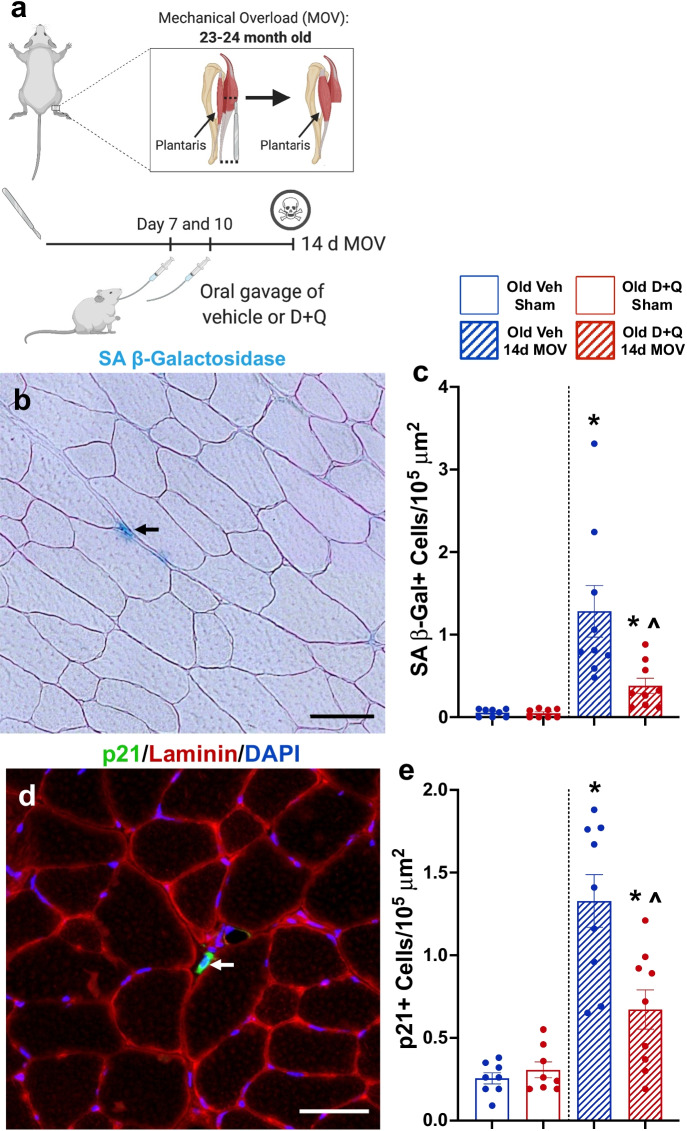
Senolytic treatment effectively cleared senescent cells in old mice following mechanical overload (MOV). (**a**) Study design schematic for the synergist ablation-induced MOV of the plantaris muscle of adult and old mice. (**b**) Representative image for senescence-associated beta-galactosidase (SA β-Gal) staining. (**c**) Quantification of SA β-Gal + cells per 10^5^ μm^2^ in old vehicle sham (open blue bar), old D + Q sham (open red bar), old vehicle 14-day MOV (hashed blue bar), and old D + Q 14-day MOV (hashed red bar). *N* = 7–9/group. (**d**) Representative image of p21 immunohistochemistry in the plantaris 14 days following MOV in a vehicle-treated old mouse. (f) Quantification of p21 + cells per 10^5^ μm^2^ in old vehicle sham (open blue bar), old D + Q sham (open red bar), old vehicle 14-day MOV (hashed blue bar), and old D + Q 14-day MOV (hashed red bar). *N* = 7–9/group. *Significance versus sham controls for a given treatment group. ^Significance versus vehicle-treated mice for a given time point

**Fig. 5:**
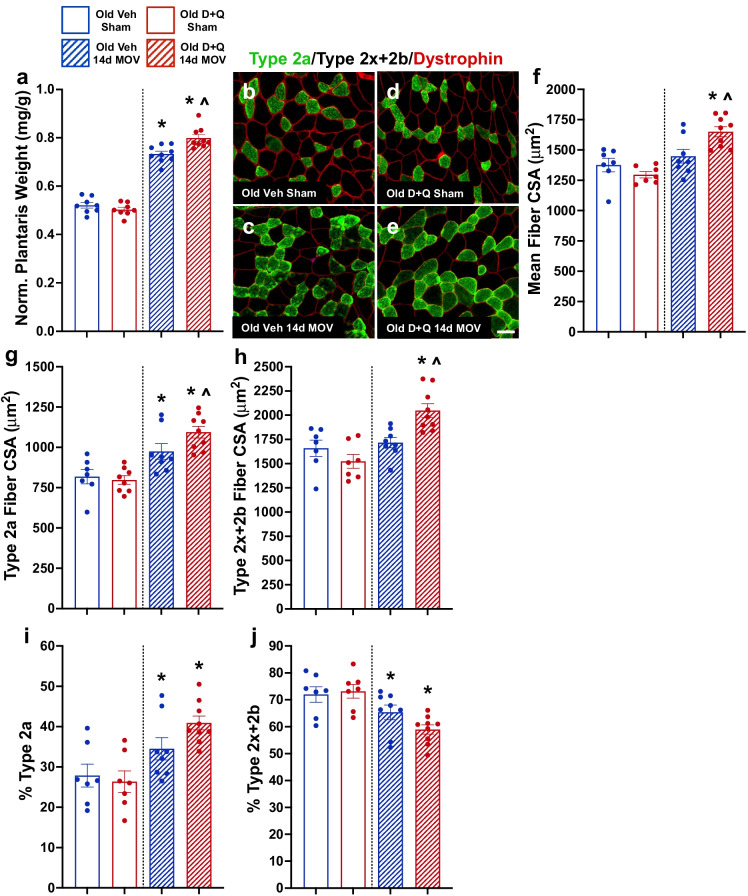
Senolytic treatment enhanced hypertrophy in response to mechanical overload (MOV) in plantaris muscles in old mice. (**a**) Normalized muscle weight in old vehicle sham (open blue bar), old D + Q sham (open red bar), old vehicle 14-day MOV (hashed blue bar), and old D + Q 14-day MOV (hashed red bar) mice. Representative images for fiber type-specific cross-sectional area (CSA) in old vehicle- (**b**, **c**) and old D + Q- (**d**, **e**) treated mice. (**f**) Mean, (**g**) type 2a, and (**h**) type 2x + 2b muscle fiber CSA in old vehicle sham, old D + Q sham, old vehicle 14-day MOV, and old D + Q 14-day MOV mice. Percent fiber-type distribution for (**i**) type 2a and (**j**) type 2x + 2b fibers. *N* = 7–9/group. *Significance versus sham controls for a given treatment group. ^Significance versus vehicle-treated mice for a given time point

Since senescent cells with MOV were most prevalent in muscle from old mice concomitant with blunted hypertrophy, we treated sham and 14-day MOV old mice with D + Q or vehicle at days 7 and 10 to see if clearance of senescent cells would promote greater muscle hypertrophy (experimental design, Fig. 4a). D + Q effectively reduced the number of senescent cells following 14 days of MOV (Fig. 4b–e), and absolute (Supplemental Fig. 2d) and normalized (Fig. 5a) muscle mass were significantly heavier in senolytic-treated compared to vehicle-treated mice, with no difference in body weight between any of the groups (data not shown). Mean muscle fiber CSA was larger after 14 days of MOV in D + Q-treated mice when compared to sham controls and vehicle-treated MOV mice (Fig. 5b–f). Both vehicle- and D + Q-treated mice had similar gains in type 2a fiber CSA (Fig. 5g); however, type 2x + 2b fibers hypertrophied only in D + Q-treated mice (Fig. 5h). There was a glycolytic to oxidative fiber-type shift in response to MOV, with no effect of D + Q (Fig. 5i, j). Therefore, clearance of senescent cells associated with gains in muscle mass and muscle fiber CSA, specifically in type 2x + 2b muscle fibers, that are most deleteriously affected by aging.

### Changes in whole-muscle gene expression in response to MOV with and without D + Q

**Fig. 6:**
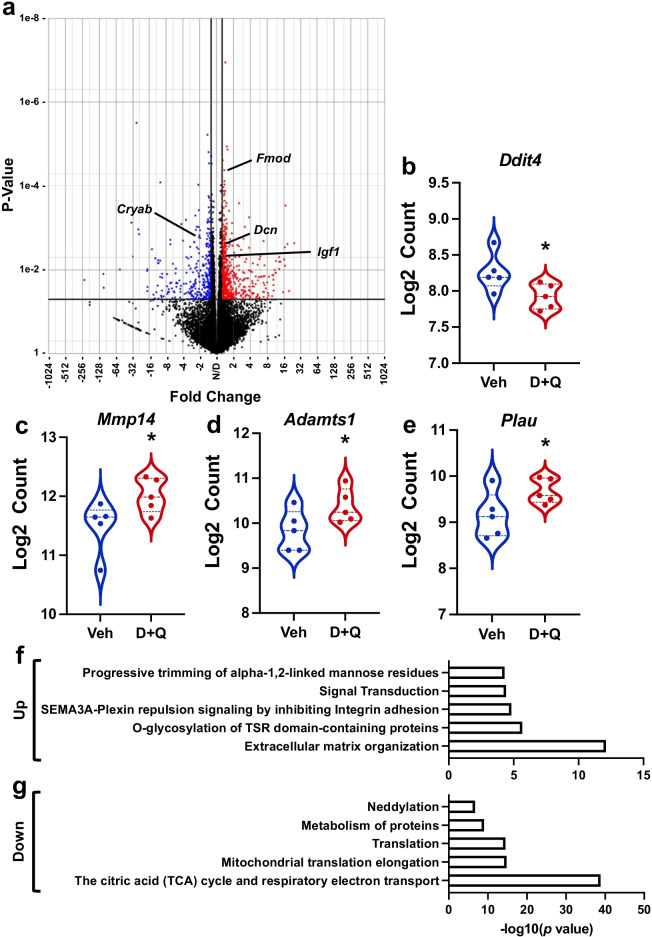
Senolytic treatment enhanced pro-growth gene expression in the plantaris muscle in response to mechanical overload (MOV) identified by RNA-sequencing. (**a**) Volcano plot showing significantly upregulated (red circles) and downregulated (blue circles) genes in D + Q- compared to vehicle-treated old mice. (**b**) Violin plot of *Ddit4* expression in response to D + Q treatment. (**c**) Violin plot of *Mmp14* expression in response to D + Q treatment. (**d**) Violin plot of *Adamts1* expression in response to D + Q treatment. (**e**) Violin plot of *Plau* expression in response to D + Q treatment. *N* = 5/group. *p < 0.05

We performed RNA sequencing on plantaris muscles from vehicle- and D + Q-treated old mice following 14 days of MOV to identify potential mechanisms regulating augmented growth in response to D + Q. We identified approximately 2,000 differentially expressed genes (DEGs; Fig. 6a) in senolytic- vs. vehicle-treated muscle. Pathway analysis revealed the most downregulated pathways were associated with the TCA cycle and electron transport chain (Supplemental Fig. 3a), while ECM remodeling and collagen degradation were some of the most upregulated pathways (Supplemental Fig. 3b). Unexpectedly, we did not see any changes in inflammatory pathways or genes; however, included in the list of downregulated genes were *Ddit4* (Fig. 6b), a potent mTORC1 inhibitor [48, 49] that, when knocked out, augments MOV-mediated hypertrophy [50], and *Cryab* (Fig. 6a), an apoptosis inhibitor [51, 52] that could play a role in the removal of senescent cells by affecting SCAPs [53]. Some of the most upregulated genes included 7 members of the ADAMTS family, enzymes that play a key role in the remodeling of the extracellular matrix (ECM) [54], myostatin regulatory genes, *Fmod* [55, 56] and *Dcn* [57, 58], and pro-growth gene, *Igf1* [59] (Fig. 6a). There was also higher expression of macrophage-secreted growth factors, such as *Mmp14* (Fig. 6c) [60], *Adamts1* (Fig. 6d) [61], and *Plau* (Fig. 6e) [62]. Due to redundancies in the pathways identified by over-representation analysis (Supplemental Fig. 3), we have compiled a summarized list of up- (Fig. 6f) and downregulated (Fig. 6g) pathways.

## Discussion

Many older adults suffer from a diminished ability to grow skeletal muscle with resistance training, termed anabolic resistance, which may be due to a host of defective biologic processes [4, 6, 63]. Unfortunately, there has been limited success in developing therapeutic interventions to augment gains in muscle mass in these individuals. Here we report that senescent cells emerge in response to a bout of resistance exercise in humans, and a larger abundance of senescent cells persist in old vs. adult mouse muscle following a hypertrophic stimulus, potentially contributing to aging-mediated anabolic resistance. This idea is supported by the observation that D + Q reduces the number of senescent cells and enhances muscle growth in old mice in response to MOV.

There are conflicting reports on the presence of senescent cells in aged skeletal muscle in vivo under resting conditions [23, 64]; however, there is a growing body of literature showing little-to-no change in the skeletal muscle senescent cell burden [23–25] or the expression of hallmark senescence markers [26, 65] with age. In the present study, we show no difference in the relative abundance of senescent cells between adult and old sham control muscles, which is consistent with our previous reports in mice [9] and humans [23]. The lack of senescent cells in resting muscle can likely be attributed to several factors. First, skeletal muscle is a primarily post-mitotic tissue and myonuclei are in a permanent state of cell cycle arrest irrespective of upregulation in the expression of cell cycle inhibitors. Second, the resident stem cell population, satellite cells, maintains a predominately quiescent state [66, 67]. Satellite cells generally only proliferate following muscle damage or in response to exercise [37, 68, 69], resulting in relatively few cell divisions across the lifespan when compared to other cell populations such as fibroblasts and endothelial cells, and likely demonstrate a low potential for replicative senescence. Lastly, muscle contains a large number of resident macrophages [15, 70], which are partially responsible for the removal of senescent cells [71, 72]. Therefore, it stands to reason that a post-mitotic tissue, with low cell turnover and high macrophage content, would not have many senescent cells even with aging.

Although we do not see many senescent cells in resting muscle, they are elevated following muscle damage in both adult and aged muscle [22, 28–31]. As MOV [35] and resistance exercise [73] also elicit some degree of muscle damage, these cells may participate in an acute injury response that may be a normal part of muscle repair. However, over-abundance and persistence of senescent cells preferentially in old muscle may be detrimental to long-term muscle adaptation to the hypertrophic stimulus. We observed significantly more senescent cells in muscle following 14 days of MOV in old compared to adult mice associated with blunted growth in the old mice. Senescent cell abundance was reduced after treating old mice with D + Q at 7 and 10 days, resulting in augmented muscle growth following 14 days of MOV. Our previous work in a model of muscle regeneration shows that the majority of senescent cells from old mice are CD11b + macrophages, which exhibit a large upregulation of SASP genes compared to cells from young mice [74]. Therefore, we performed RNA-seq analysis on 14-day MOV muscles from vehicle- and D + Q-treated mice to determine the overall effect of senolytics relative to vehicle. Surprisingly, there was no difference in the expression of hallmark SASP genes between vehicle- and D + Q-treated mice following MOV, although the effect of D + Q on the SASP could be masked by the robust inflammatory response that is often observed following MOV [62, 75]. We observed an overall reduction in the expression of genes encoding TCA cycle and electron transport chain components, which could be beneficial within the context of the oxidative damage theory of mammalian aging [76], and elevated expression of genes involved in ECM remodeling. A time course study of the muscle transcriptomic response to MOV in young mice shows a reduction in genes associated with the TCA cycle [77], whereas ECM remodeling is a key component of hypertrophic growth [78, 79] that is defective with aging in response to resistance exercise [80, 81]. There was also lower expression of the mTORC1 inhibitor gene, *Ddit4* (REDD1), while myostatin regulatory genes, *Fmod* [55, 56] and *Dcn* [57, 58], were elevated in senolytic-treated mice. Although not quantified in this study, aberrant mTORC1 activation has been linked to sarcopenia and anabolic resistance [4, 5]. Restoration of mTORC1 signaling in old rodents restores the hypertrophic response [5], which could be mediated by a reduction in REDD1 [50]. Furthermore, there was higher expression of genes shown to be beneficial for muscle adaptation, such as *Igf1* [82], *Mmp14* [60], *Adamts1 *[61], and *Plau* [62]. Interestingly, these factors have all been shown to be secreted by M2 macrophages, which are positively associated with muscle adaptation [15, 60–62, 83]. A recent publication by our laboratory shows that M2 macrophages are the primary source of *Mmp14* mRNA in plantaris muscle following MOV [60]. Although D + Q may specifically target senescent cells, we cannot discount the potential impact of D + Q on gene expression in proliferating cell populations, including macrophages, as well as post-mitotic muscle fibers, as D + Q is non-lethal to healthy cells [18]. That D + Q has functions beyond its senolytic properties is evidenced by the fact that in vitro, D + Q increases myogenic progenitor cell proliferation [9].

The results of this study provide the first evidence showing the induction of senescent cells in human and mouse skeletal muscle in response to a hypertrophic stimulus, with old mice displaying a greater accumulation of senescent cells than young mice. Most importantly, we find that the clearance of senescent cells with D + Q restores muscle growth in old mice, suggesting the exciting possibility that senolytics may provide an effective therapeutic strategy to enhance muscle growth in old humans following resistance training.

## Supplementary Information

Below is the link to the electronic supplementary material.

Supplementary file1 Supplemental Figure 1. Cellular localization of p21 in muscle visualized using immunohistochemistry. Representative image of cytoplasmic p21 (green) outside the muscle fiber sarcolemma (red) that does not overlay with the nucleus (blue; **top panels**). Representative image of p21 (green) inside the muscle fiber sarcolemma (red) co-localized with the nucleus (blue; **bottom panels)**. Supplemental Figure 2. Additional characteristics of plantaris muscle following mechanical overload (MOV). **a)** Absolute muscle weight in adult (blue circles) and old (red triangles) mice following sham surgery (controls) and 7- and 14-days of MOV induced by synergist ablation surgery. **b)** Type 2a and **c)** Type 2x+2b fiber distribution following sham surgery (controls) and 7- and 14-days of MOV induced by synergist ablation surgery. **d)** Absolute muscle weight in old vehicle sham (open blue bar), old D+Q sham (open red bar), old vehicle 14d MOV (hashed blue bar), and old D+Q 14d MOV (hashed red bar) mice following sham surgery (controls) and 14-days of MOV induced by synergist ablation surgery. N=6-9/group. * indicates significance versus sham controls for a given treatment group. Supplemental Figure 3. Overrepresented pathway analysis of down and up regulated genes in response D+Q treatment during mechanical overload (MOV) of plantaris muscle. **a)** The 30 most down regulated pathways in order of ascending p-value. **b)** The 30 most up regulated pathways in order of ascending p-value (PPTX 3923 KB)

## Data Availability

RNA sequencing data has been deposited to NCBI Gene Expression Omnibus under accession number GSE195707.

## Code availability

N/A.
