# Supplementary material for: Senolytic treatment rescues blunted muscle hypertrophy in old mice
Source: GeroScience. 2022 Mar 24;44(4):1925–40. doi: 10.1007/s11357-022-00542-2 (PMC9616988; doi:10.1007/s11357-022-00542-2)
Supplement: Supplementary file 1 — Supplementary file1 Supplemental Figure 1. Cellular localization of p21 in muscle visualized using immunohistochemistry. Representative image of cytoplasmic p21 (green) outside the muscle fiber sarcolemma (red) that does not overlay with the nucleus (blue; top panels). Representative image of p21 (green) inside the muscle fiber sarcolemma (red) co-localized with the nucleus (blue; bottom panels). Supplemental Figure 2. Additional characteristics of plantaris muscle following mechanical overload (MOV). a) Absolute muscle weight in adult (blue circles) and old (red triangles) mice following sham surgery (controls) and 7- and 14-days of MOV induced by synergist ablation surgery. b) Type 2a and c) Type 2x+2b fiber distribution following sham surgery (controls) and 7- and 14-days of MOV induced by synergist ablation surgery. d) Absolute muscle weight in old vehicle sham (open blue bar), old D+Q sham (open red bar), old vehicle 14d MOV (hashed blue bar), and old D+Q 14d MOV (hashed red bar) mice following sham surgery (controls) and 14-days of MOV induced by synergist ablation surgery. N=6-9/group. * indicates significance versus sham controls for a given treatment group. Supplemental Figure 3. Overrepresented pathway analysis of down and up regulated genes in response D+Q treatment during mechanical overload (MOV) of plantaris muscle. a) The 30 most down regulated pathways in order of ascending p-value. b) The 30 most up regulated pathways in order of ascending p-value (PPTX 3923 KB) [file 11357_2022_542_MOESM1_ESM.pptx]

## Slide 1
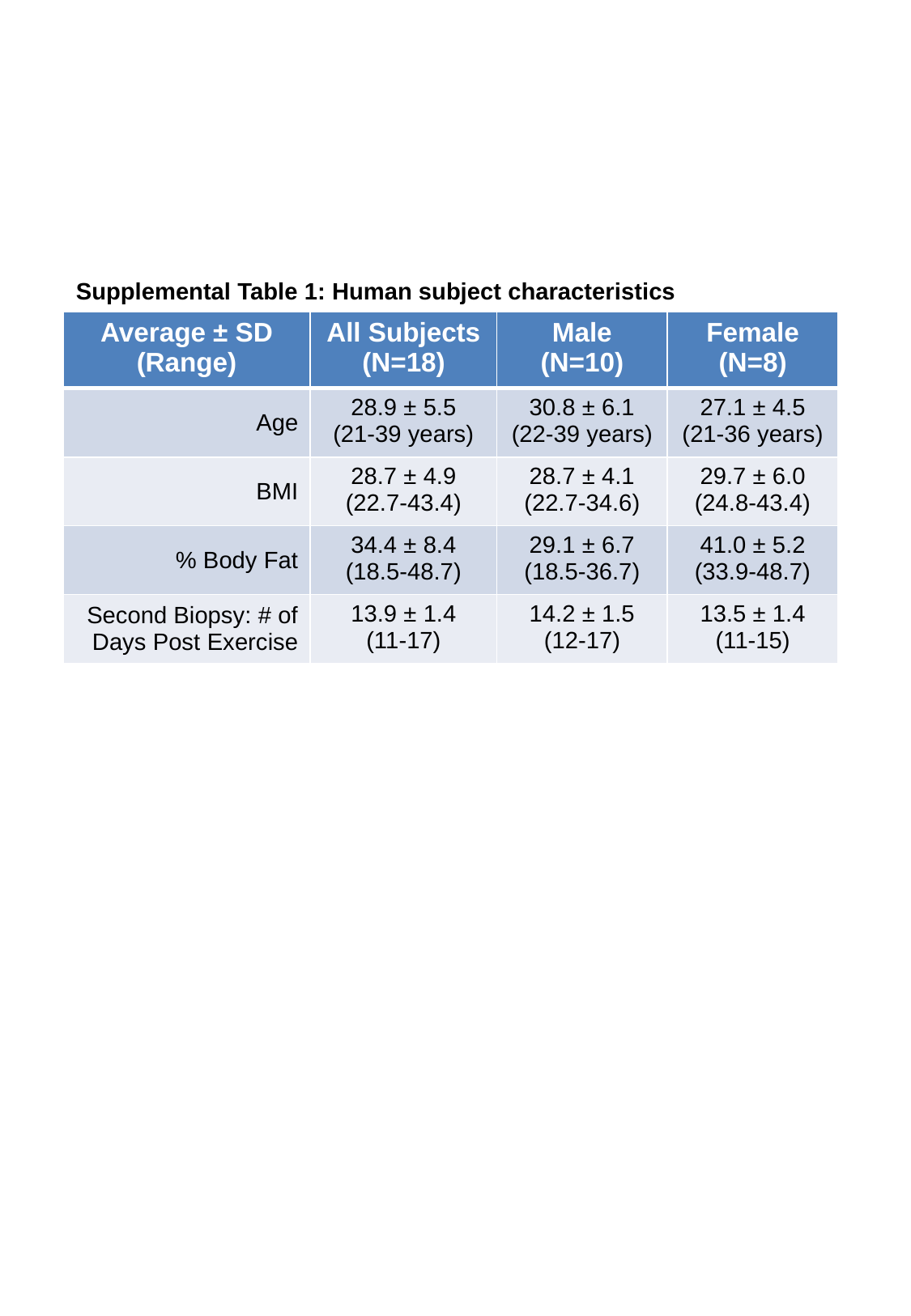

Supplemental Table 1: Human subject characteristics
| Average ± SD (Range) | All Subjects (N=18) | Male (N=10) | Female (N=8) |
| --- | --- | --- | --- |
| Age | 28.9 ± 5.5 (21-39 years) | 30.8 ± 6.1 (22-39 years) | 27.1 ± 4.5 (21-36 years) |
| BMI | 28.7 ± 4.9 (22.7-43.4) | 28.7 ± 4.1 (22.7-34.6) | 29.7 ± 6.0 (24.8-43.4) |
| % Body Fat | 34.4 ± 8.4 (18.5-48.7) | 29.1 ± 6.7 (18.5-36.7) | 41.0 ± 5.2 (33.9-48.7) |
| Second Biopsy: # of Days Post Exercise | 13.9 ± 1.4 (11-17) | 14.2 ± 1.5 (12-17) | 13.5 ± 1.4 (11-15) |

## Slide 2
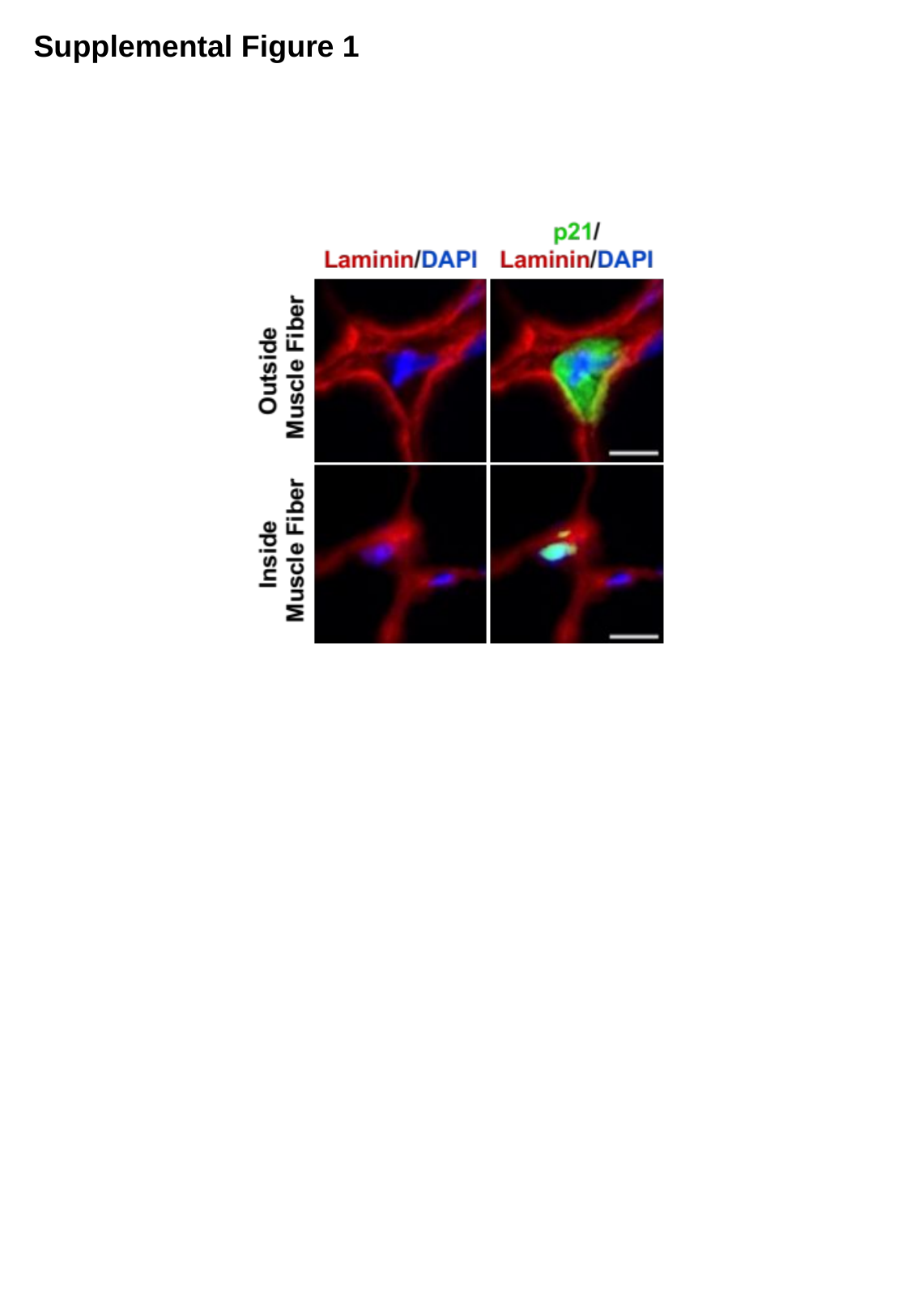

Supplemental Figure 1

## Slide 3
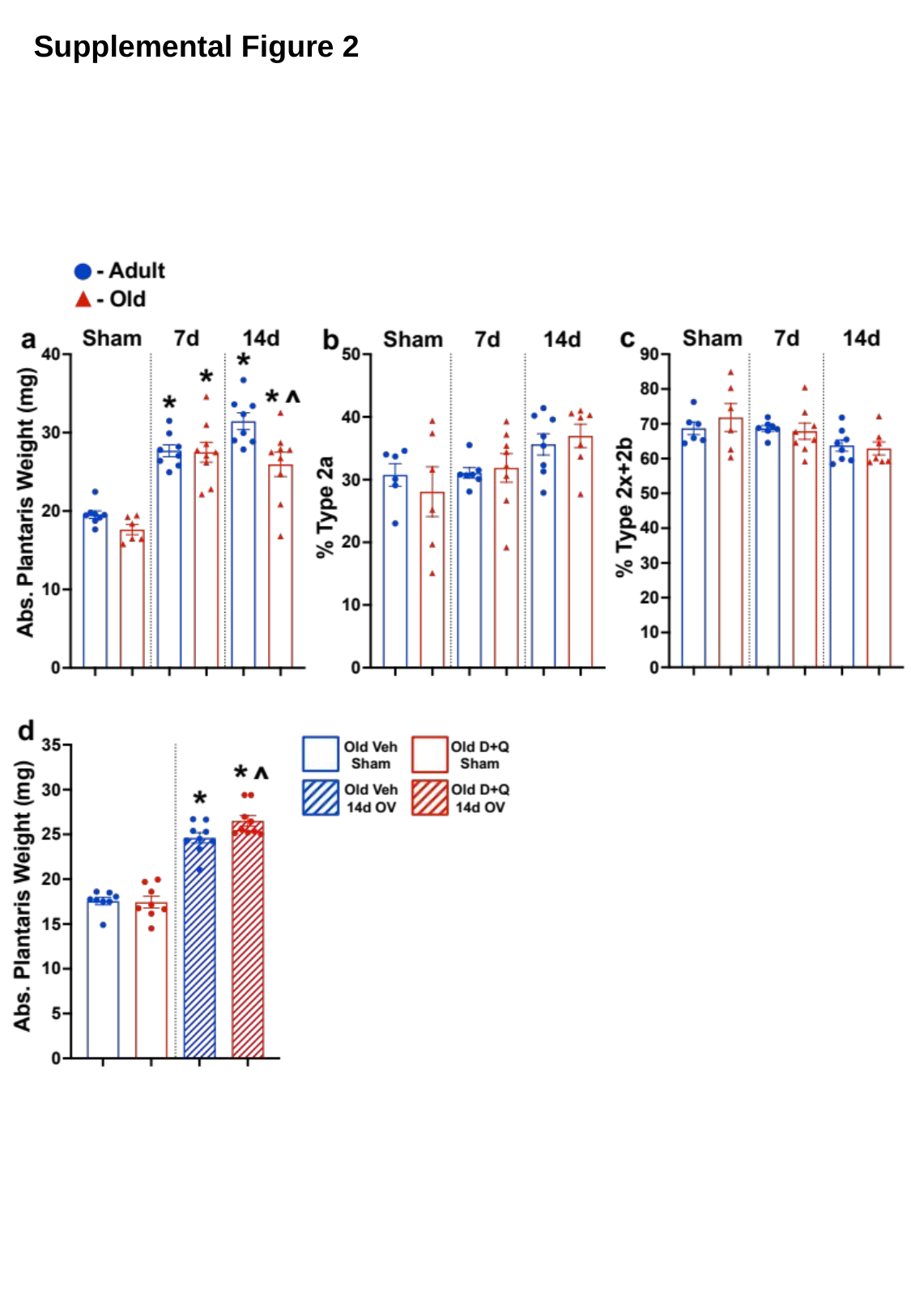

Supplemental Figure 2

## Slide 4
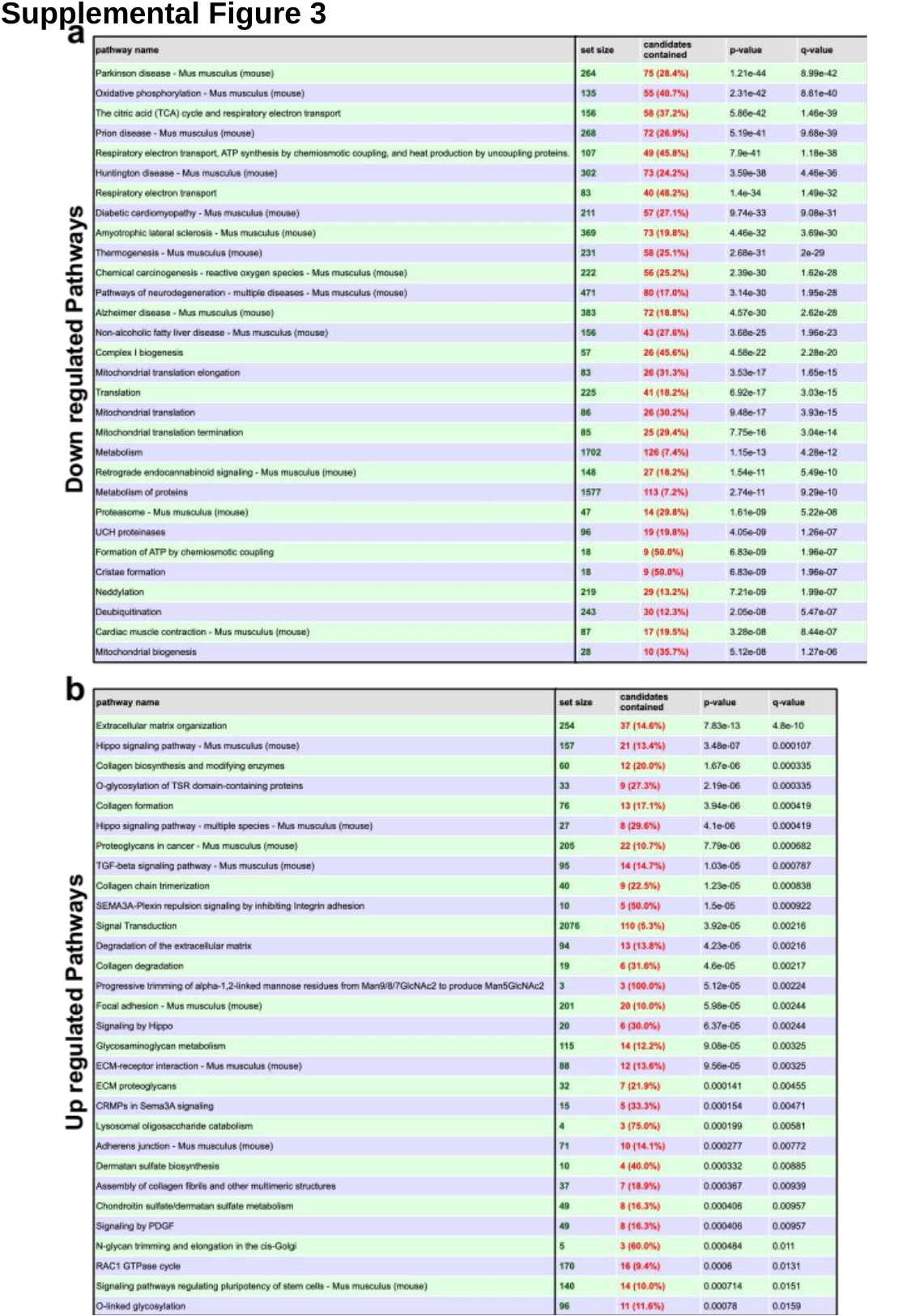

Supplemental Figure 3
